# A Diagnostic Algorithm Based on Urine Antigen and Blood Polymerase Chain Reaction Testing for Visceral Leishmaniasis in HIV-Coinfected Patients in Northwest Ethiopia

**DOI:** 10.4269/ajtmh.26-0104

**Published:** 2026-09-15

**Authors:** Ermias Diro, Koert Ritmeijer, Saskia van Henten, Aderajew Kibret, Mekibib Kassa, Hailemariam Beyene, Wim Adriaensen, Myrthe Pareyn, Johan van Griensven

**Affiliations:** ^1^ Department of General Internal Medicine, University of Washington, Seattle, Washington, USA;; ^2^ Médecins sans Frontières, Amsterdam, The Netherlands;; ^3^ Institute of Tropical Medicine, Antwerp, Belgium;; ^4^ Médecins sans Frontières, Abdurafi, Ethiopia;; ^5^ Leishmaniasis Research and Treatment Center, University of Gondar, Gondar, Ethiopia

## Abstract

The diagnosis of visceral leishmaniasis (VL) in HIV patients relies on tissue aspiration, which is potentially life-threatening. Because antibody-based tests are unreliable for this group, *Leishmania* blood polymerase chain reaction (PCR) and urine antigen tests can serve as noninvasive alternatives. Using data from the PreLeisH cohort (ClinicalTrials.gov [NCT03013673]), the diagnostic accuracy of these two tests was determined individually, and an algorithm was designed for targeted tissue aspiration. Of the 490 PreLeish participants, 51 underwent diagnostic procedures for VL 118 times at different follow-up time points when clinically suspected. Of these, 48 (94.1%) patients with 102 (84.4%) procedures had complete data, including tissue aspiration, *Leishmania* kinetoplast DNA blood PCR, and urine antigen (KAtex [Kalon Biological Ltd., Guildford, United Kingdom]) results. The diagnostic performance of KAtex and blood PCR testing was assessed individually against tissue aspiration, and sequential and parallel algorithms were developed. Visceral leishmaniasis was confirmed via tissue microscopy in 55/102 (54%) episodes. The sensitivity and specificity of blood PCR testing were 98% and 64%, respectively; the sensitivity and specificity of KAtex testing were 93% and 81%, respectively. A sequential algorithm using KAtex first and restricting PCR to KAtex-negative suspects limited PCR testing to 42 (41.2%) episodes and tissue aspiration to 14 (13.7%) episodes, with sensitivity, specificity, and positive predictive value of 100%, 81%, and 86%, respectively. The parallel testing algorithm exhibited comparable performance but requires PCR testing for all suspects. The sequential KAtex and PCR testing algorithm, combined with targeted tissue aspiration, met the WHO target product profile, except for specificity. This approach supports efficient resource use and reduces the need for invasive procedures.

## INTRODUCTION

Visceral leishmaniasis (VL) is a protozoan infection transmitted by sandflies. The infection targets the reticuloendothelial system, causing chronic fever, weight loss, splenomegaly, and pancytopenia, and is fatal if untreated.^1^ It is caused by parasites belonging to the *Leishmania donovani* (*L. donovani*) complex. In Latin America and the Mediterranean region, VL is caused by *Leishmania infantum* and has zoonotic transmission, whereas in the Indian subcontinent and East Africa, it is caused by *L. donovani*, and transmission is anthroponotic. Globally, ∼50,000 to 90,000 cases occur annually.^2^ Historically, the Indian subcontinent used to have the highest disease burden. In the last decade, after a successful elimination program was implemented in the Indian subcontinent, the East Africa region now has the highest disease burden.^3^^,^^4^

The WHO recently launched a strategic framework to eliminate VL as a public health problem in Eastern Africa.^5^ Reducing the case fatality of primary VL to below 1% is proposed as a key indicator of program success. Other targets include early VL case detection and HIV diagnosis and co-management. Visceral leishmaniasis/HIV-coinfected individuals face recurrent relapses and a high case fatality rate, and because they might be more infectious, they could drive VL transmission.^6^ Consequently, the WHO framework stipulates the need for strategies to improve access to early diagnosis and treatment of VL/HIV coinfection.^5^ Although the rK39 rapid diagnostic test (RDT) constitutes the backbone of VL diagnosis, it has suboptimal performance in Eastern Africa.^7^ Additionally, antibody-based serological tests have been found to be even less sensitive and specific in HIV-infected individuals and not useful for diagnosing relapses.^8^^,^^9^ Thus, a shift to using antigen and molecular testing represents a significant opportunity.

In the Northwest part of Ethiopia, 10% to 20% of VL patients have HIV coinfection.^10^ Consequently, VL diagnosis relies on the microscopic detection of parasites via spleen or bone marrow aspiration. Spleen aspiration carries a risk of fatal splenic bleeding and requires skilled manpower to conduct the procedure. As a result, it is limited to specialized referral centers where surgical and blood transfusion services are available to manage potential complications. Bone marrow aspiration is painful and requires local anesthetics, a bone marrow set (special needle), and expertise. Therefore, there is a crucial need for reliable noninvasive diagnostics that can ideally be used in VL-endemic areas where resources and skilled manpower are limited. In line with this need, the WHO has called for noninvasive tests to diagnose VL and assess cure status in HIV-infected individuals.^11^ The target product profile for the diagnosis of VL stipulates a minimum sensitivity of 95%, a specificity of 96.5%, a positive predictive value (PPV) of 75%, and a negative predictive value (NPV) of 95%, including for HIV-coinfected individuals.^12^

In a previous study of HIV-coinfected patients, the KAtex (Kalon Biological Ltd., Guildford, United Kingdom) urine antigen test had moderate sensitivity (82%) but limited specificity (49%).^13^^,^^14^ Notably, with expanding HIV and tuberculosis programs and after the coronavirus disease 2019 (COVID-19) pandemic, availability and experience in molecular diagnostics such as polymerase chain reaction (PCR) testing are increasing, including in VL-endemic settings in East Africa. Thus, it is high time to assess the utility of these diagnostics for VL in HIV patients in whom antibody-based serology tests such as rK39 have poor performance. The combined use of urine antigen and molecular diagnostics has not been studied previously. In the present study, a diagnostic algorithm using KAtex and blood PCR tests was explored for VL diagnosis in HIV-coinfected individuals.

## MATERIALS AND METHODS

### Study design and aim.

The present prospective cohort study is nested within the PreLeisH study (ClinicalTrials.gov (NCT03013673).^15^ The main objective of the PreLeisH study was to identify *Leishmania* markers predictive of VL in HIV-infected individuals living in a VL-endemic area; the details of the study have been described elsewhere.^15^

In the present study, the diagnostic accuracy of KAtex and blood PCR testing for diagnosing VL in HIV-infected patients was individually assessed against tissue aspiration microscopy. Sequential and parallel testing algorithms were developed and assessed with the aim to design an approach that can reduce the number of PCR tests for cost and logistical reasons and minimize the number of patients who require tissue aspiration for safety reasons, while meeting WHO target product expectations.

### Study site and period.

The present study was conducted at the Abdurafi Health Centre, located in a VL-endemic area in Northwest Ethiopia, between October 2017 and May 2021. Routine care for VL patients at the study site is supported by Médecins sans Frontières. Patients were followed for 2 years.

### Study population.

HIV-infected adults in the Abdurafi HIV care program were prospectively enrolled in the PreLeisH study. In this cohort, patients underwent diagnostic workup when they were suspected of having VL disease. Those with complete KAtex, blood PCR, and tissue aspiration results were included in the present sub-study.

### Study procedure.

Clinicians evaluated patients for VL when they suspected it (based on typical or atypical manifestations) at each study visit (every 3–6 months) or during unscheduled visits. Those with clinical suspicion of VL underwent diagnostic work-up. Visceral leishmaniasis diagnosis was based on the microscopic detection of *Leishmania* parasites in spleen or bone marrow aspirates. Additionally, a real-time PCR test (referred to as PCR in this text) targeting *Leishmania* kinetoplast DNA (kDNA) on venous blood samples and a *Leishmania* urine antigen test (KAtex) were performed. Visceral leishmaniasis diagnosis was made independently of KAtex and PCR test results because samples were transported in a cold chain to the Leishmaniasis Research and Treatment Center in Gondar and processed in batches; therefore, results were not immediately available.

Urine samples were aliquoted within 3 hours after collection and kept at −40°C until analysis. The KAtex *Leishmania* urine antigen test detects a low-molecular-weight *Leishmania* antigen via agglutination with polystyrene latex particles coated with polyclonal anti-*L. donovani* antigen antibodies.^16^^,^^17^ It is a semi-quantitative urine assay, with three levels of agglutination reaction scored as 1+ (weakly positive), 2+ (moderately positive), or 3+ (strongly positive). Agglutination of any degree visible to the naked eye was considered positive, whereas no agglutination was considered negative.

The PCR test targeting kDNA on whole blood has been described previously.^18^^,^^19^ Samples were run with a negative extraction and PCR control in duplicate to check for contamination. For samples with cycle threshold (Ct) values >35, extraction and PCR testing were repeated to confirm positivity. A PCR test result was considered positive if the Ct value was <35, or positive twice with a value >35 in two separate extractions. The Ct value from the first run was used for analysis in case of duplicate runs.

### Monitoring and quality control.

All tests were conducted following test-specific standard operating procedures developed in accordance with the manufacturer’s guidelines. The Institute of Tropical Medicine, Antwerp (ITM-A), Clinical Trials Unit provided support in the set-up, implementation, and monitoring of the study. The ITM-A Clinical Reference Laboratory supported all laboratory procedures, including assay set-up and validation, establishment of the laboratory analytical plan, quality control, and on-site training.

## STATISTICAL ANALYSES

The diagnostic performance of KAtex and blood PCR testing was assessed separately by calculating the sensitivity, specificity, PPV, and NPV using tissue aspirate microscopy as a standard. Subsequently, the utility of sequential and parallel diagnostic designs using KAtex, blood PCR testing, and tissue aspiration was assessed in terms of meeting the WHO target product profile and supporting treatment decision-making. These approaches were compared by the proportion of individuals who required PCR testing and tissue aspiration.

The sequential diagnostic algorithm (Figure 2) followed a similar approach to the algorithm used in East African national guidelines for primary VL in HIV-negative patients.^20^ In this existing algorithm, VL suspects are first tested with a simple and cheap test, the rK39 RDT. A positive rK39 RDT result allows for the diagnosis of VL; those with a negative rK39 RDT result are subsequently tested with a more expensive and complex test, the direct agglutination test (DAT). With high-titer DAT results, VL is ruled in; with a negative test result, VL is ruled out. Only those with borderline DAT titers undergo tissue aspiration, which is an invasive test.

When adopting similar sequential diagnostic algorithm principles for HIV-coinfected individuals, KAtex, which is cheaper and easier to perform, was used as the first test; blood PCR testing was used as the second test; and tissue aspiration was used as the final test, if indicated. This approach was used to allow for the rational use of available resources and minimize the invasive tissue aspiration procedure while targeting the WHO target product profile of PPV ≥75% and NPV ≥95%.^12^ In the sequential algorithm approach, if diagnostic performance remained below the target PPV or NPV after the first test, a second test had to be performed. In the parallel testing algorithm, both KAtex and blood PCR testing were performed at the same time, followed by tissue aspiration in case of discordances.

Exploratory analysis using semi-quantitative categories for the individual tests, such as classifying the KAtex titer result as negative, 1+/2+, or 3+ and subcategorizing blood PCR results as negative, low Ct (<25), or high Ct (≥25), was also conducted to identify alternative approaches to meeting the WHO target product specificity (96.5%).

## RESULTS

Of the 490 HIV-infected individuals enrolled in the PreLeish study at Abdurafi Health Center in a VL-endemic area in Northwest Ethiopia, 51 underwent tissue aspiration at least once, for a total of 118 suspected VL episodes over a 2-year follow-up period. Data from 16 episodes were excluded because results were missing for either blood PCR testing or urine KAtex, leaving 48 individuals with 102 episodes for analysis (Figure 1, diagram of inclusion and exclusion).

**Figure 1.**
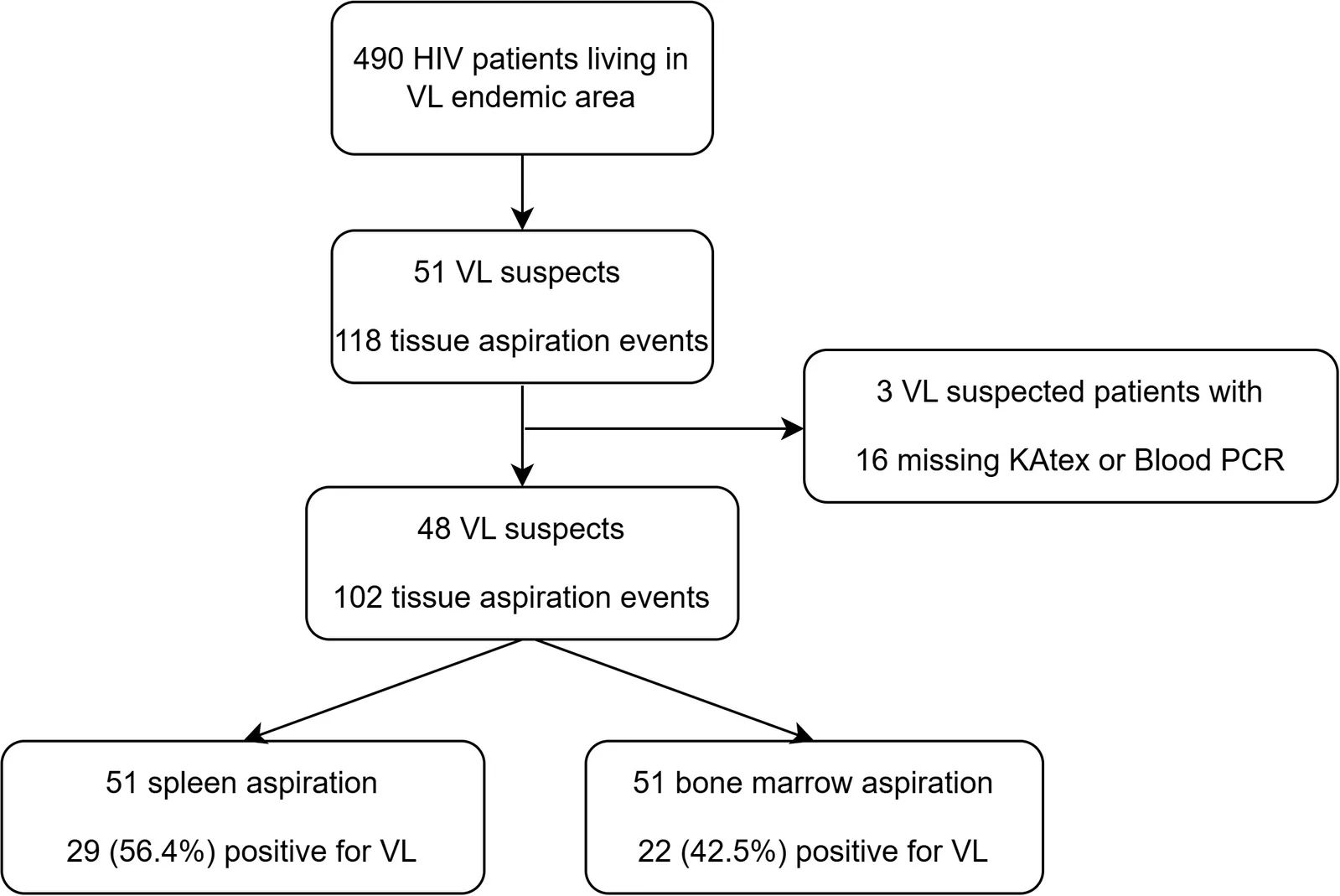
Study recruitment flow diagram. VL = visceral leishmaniasis.

All participants were male with a median age of 35.5 years (30.0–41.5 years). All but one were on antiretroviral therapy at enrollment; the median cluster of differentiation 4 count was 207 cells/*µ*L (interquartile range [IQR]: 85–379 cells/*µ*L). A history of VL was recorded for 34 (71%) participants, with 20 (41.7) having a VL episode within the last year before study enrollment (Table 1).

**Table 1:** Baseline characteristics of the study population

Baseline Characteristics	Value, *N* = 48
Age (years)	35.5 (30.0–41.5)
Sex, male (%)	48 (100)
Baseline CD4 count (*n* = 47), median (IQR)	207 (85–379)
Previous VL episodes (at baseline)	
None	14 (29.2)
≥1 year before	14 (29.2)
<1 year before	20 (41.7)
On ART at study enrollment, *n* (%)	47 (98.0)
VL diagnostic procedures	
Spleen, *n* (%)	51 (50)
Bone marrow, *n* (%)	51 (50)

ART = antiretroviral treatment; CD4 = cluster of differentiation 4; IQR = interquartile range; VL = visceral leishmaniasis.

The total tissue aspiration count per patient ranged from one to seven because the procedure was performed each time a patient was clinically suspected of having VL disease during the 2-year follow-up period. Twenty-five (52%) patients underwent tissue aspiration more than once, and overall, the median number of tissue aspirations per patient during the follow-up period was two (IQR: 1–3). Half (*n* = 51) of the tissue aspirations were performed on the spleen, and the remaining half were performed on bone marrow. Of 102 tissue aspirates, *Leishmania* parasites were detected via microscopy in 55 (53.9%), with a positive rate of 42.5% in bone marrow aspirates and 56.4% in spleen aspirates.

Urine KAtex had a sensitivity of 92.7% and a specificity of 80.9% in diagnosing VL, with a PPV of 85%. Blood PCR testing had a sensitivity of 98.2% and a specificity of 63.8%, with a PPV of 76.1% and an NPV of 96.8% (Table 2). In a subgroup analysis, specificity was higher in splenic aspirates compared with bone marrow samples (75% versus 56% for PCR and 95% versus 70% for KAtex), whereas sensitivity was comparable (97% versus 100% for PCR and 92% versus 94% for KAtex).

**Table 2:** Diagnostic performance of KAtex and blood polymerase chain reaction testing for visceral leishmaniasis among HIV-infected patients compared with tissue aspiration microscopy

Diagnostic tests and results	Tissue Aspiration		Total	Sensitivity	Specificity	Predictive Value	
	Positive	Negative				Positive	Negative
KAtex-positive	51	9	60	92.7	80.9	85.0	90.5
KAtex-negative	4	38	42	–	–	–	–
Total	55	47	102	–	–	–	–
Blood PCR-positive	54	17	71	98.2	63.8	76.1	96.8
Blood PCR-negative	1	30	31	–	–	–	–
Total	55	47	102	–	–	–	–

PCR = polymerase chain reaction; VL = visceral leishmaniasis. The performance of sequential and parallel testing algorithms was assessed on the basis of these findings.

In the sequential testing algorithm, if a clinically suspected patient tested positive via KAtex, the PPV was 85% (above the WHO PPV target of ≥75%); therefore, VL treatment could be started without additional testing. Those who tested negative on KAtex needed another test because the NPV was below the WHO target NPV (≥95%). A negative PCR result in KAtex-negative patients exhibited a 100% NPV. These patients needed to be evaluated for alternative pathologies other than VL. For patients with a positive PCR result after a negative KAtex test result, the PPV was 28.6%; therefore, they needed a tie-breaker test: tissue aspiration. In this sequential diagnostic algorithm approach, 42/102 (41.2%) patients needed PCR testing, and only 14/102 (13.7%) needed tissue aspiration. This algorithm exhibited 100% sensitivity, 81.0% specificity, 100% NPV, and 85.9% PPV for diagnosing VL in HIV patients (Figure 2; Table 3).

**Figure 2.**
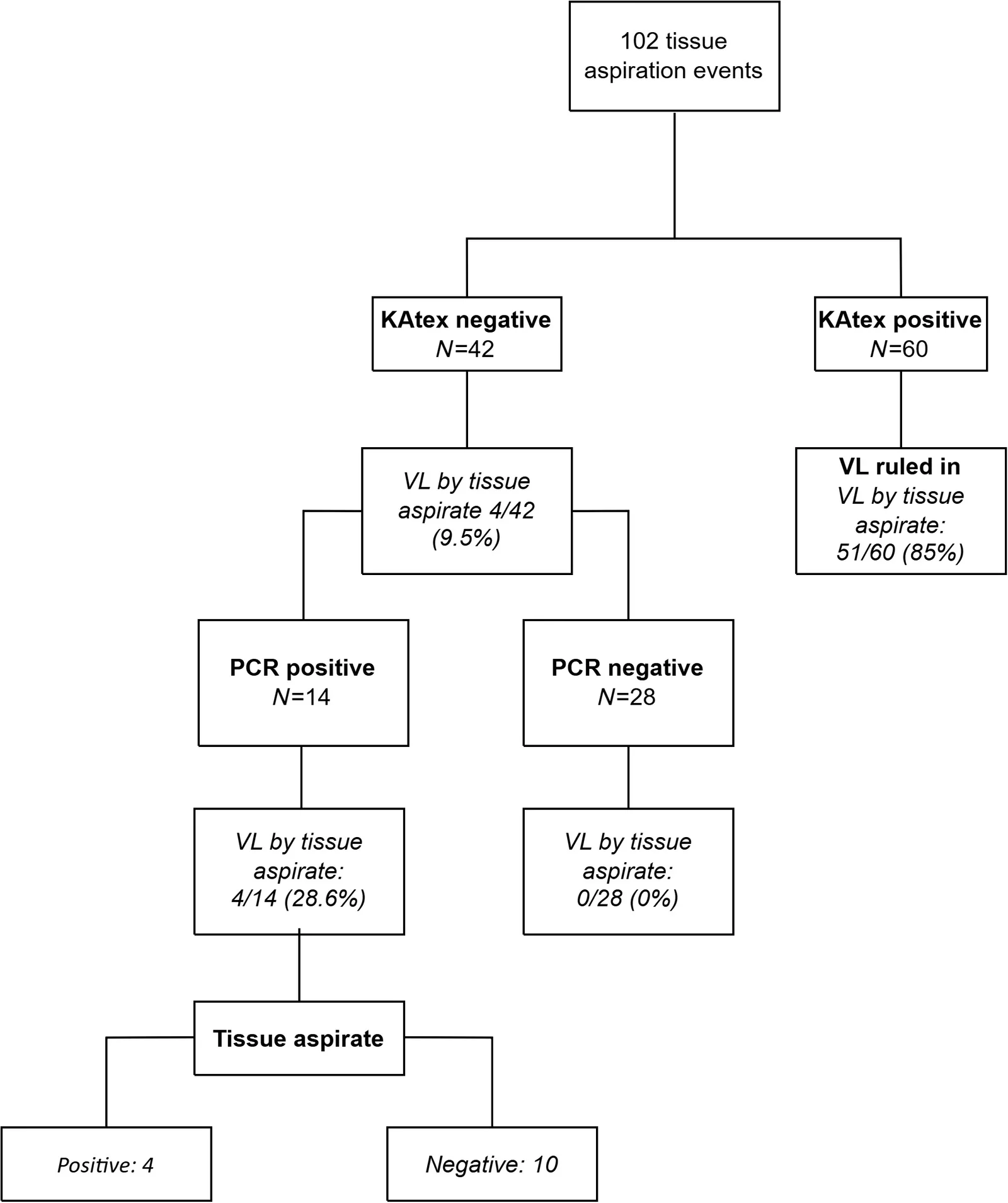
Algorithm to diagnose visceral leishmaniasis in Ethiopian HIV-infected individuals sequentially using KAtex and blood polymerase chain reaction testing. VL = visceral leishmaniasis.

**Table 3:** Diagnostic accuracy of *Leishmania* blood polymerase chain reaction and urine antigen tests and a sequential testing algorithm to diagnose visceral leishmaniasis in Ethiopian HIV-infected individuals

Diagnostic tests and their performance	KAtex, % (*n/N*)	PCR, % (*n/N*)	KAtex Followed by PCR (sequential), % (*n*/*N*)	KAtex and PCR in Parallel, % (*n*/*N*)	Using Semi-Quantitative KAtex and PCR (sequential), % (*n*/*N*)*
Sensitivity	92.7 (51/55)	98.2 (54/55)	100 (55/55)	100 (55/55)	100 (55/55)
Specificity	80.9 (38/47)	63.8 (30/47)	81 (38/47)	85 (40/47)	91.5 (43/47)
PPV	85 (51/60)	76.1 (54/71)	85.9 (55/64)	88.7 (55/62)	93.2 (55/59)
NPV	90.5 (38/42)	96.8 (30/31)	100 (38/38)	100 (40/40)	100 (0/43)
Tests performed					
KAtex	100 (102/102)	–	100 (102/102)	100 (102/102)	100 (102/102)
PCR	–	100 (102/102)	41.2 (42/102)	100 (102/102)	52.9 (54/102)
Tissue aspiration	–	–	13.7 (14/102)	16.7 (17/102)	18.6 (19/102)

PCR = polymerase chain reaction; PPV = positive predictive value; NPV = negative predictive value.

* Results from an exploratory analysis using categories of KAtex and PCR results.

A parallel testing algorithm (testing all VL suspects with both KAtex and blood PCR at the same time, followed by tissue aspiration in case of discrepancy) yielded a comparable diagnostic accuracy to the sequential testing; however, it required blood PCR testing to be performed in all episodes and tissue aspiration to be performed in 17 (16.7%) episodes (Figure 3; Table 3).

**Figure 3.**
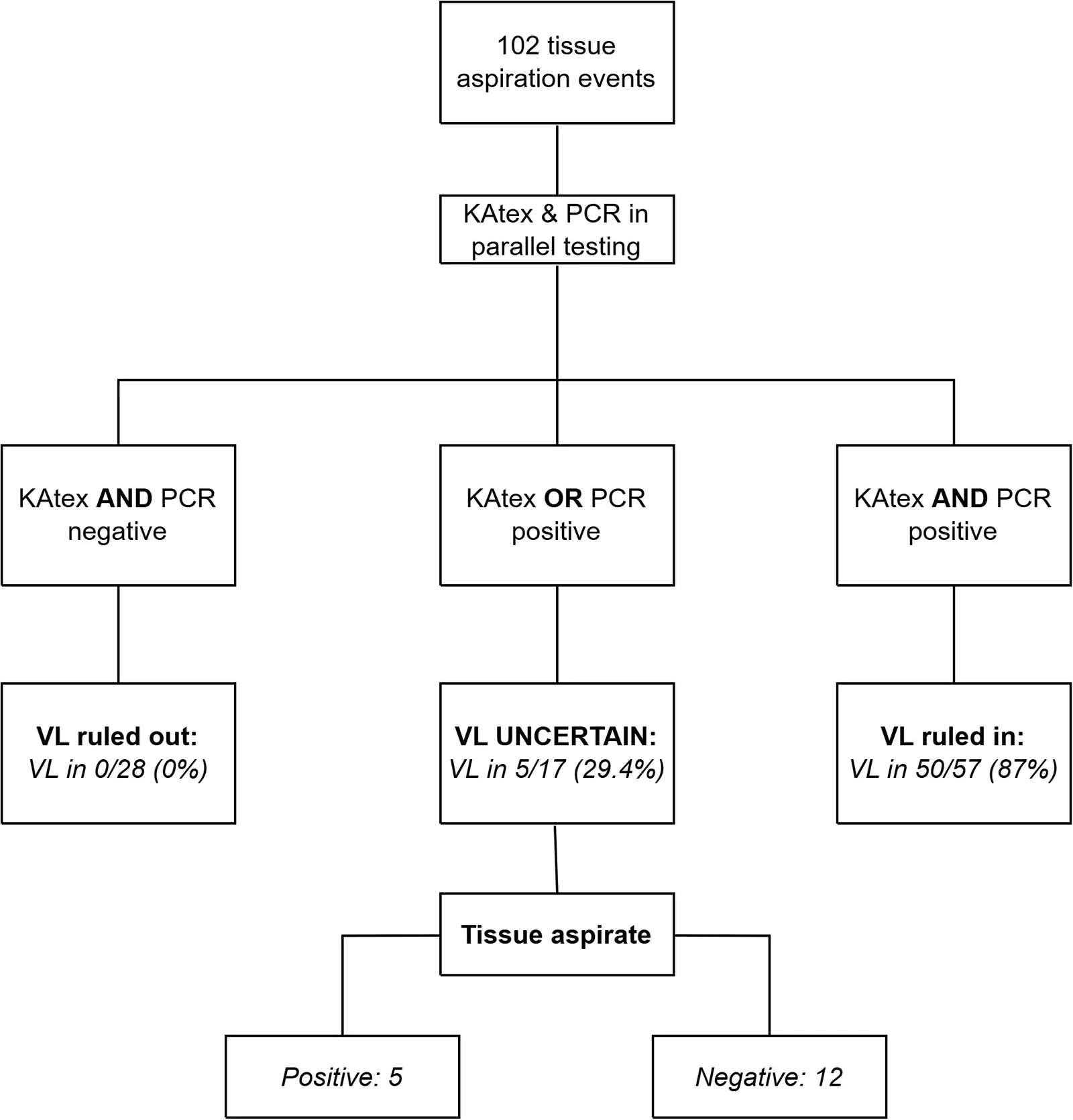
Algorithm to diagnose visceral leishmaniasis in Ethiopian HIV-infected individuals using KAtex and blood polymerase chain reaction testing in parallel. VL = visceral leishmaniasis.

An exploratory analysis was conducted using semi-quantitative KAtex and PCR results to improve algorithm specificity. KAtex results categorized into semi-quantitative groups (0, +1/+2, +3) were followed by PCR testing, which was also grouped on the basis of Ct values (negative, <25 versus ≥25; Supplemental Figures 1 and 2). With this sequential approach, the algorithm’s specificity increased to 91.5%, and the PPV increased to 93.2%; however, 54 (52.9%) patients required PCR testing. The algorithm using three KAtex result categories resulted in some groups with very small sample sizes (Supplemental Figure 1). Merging the lower two categories (0 and 1+/2+) yielded the same diagnostic accuracy while increasing simplicity (Supplemental Figure 2).

## DISCUSSION

In the VL elimination effort in East Africa, diagnostics with good performance that can be used at decentralized care levels are paramount.^5^ In this region, where the HIV coinfection rate is high and poses a diagnostic challenge, the utility of a urine antigen-based (KAtex) and molecular (blood PCR) tests was explored among HIV infected patients suspected of VL in the present study. The authors used an algorithm aimed at achieving the WHO diagnostic target product profile while promoting efficient resource use and reducing the frequency of invasive procedures (tissue aspiration).^12^

When assessed separately, the performance of blood PCR testing meets the WHO target product profile expectations for PPV (76%), NPV (98%), and sensitivity (98%), but the specificity (64%) is suboptimal. The low specificity could be partially due to the low sensitivity of tissue aspiration used as a reference test.^21^ On the other hand, KAtex meets the target product PPV (85%), although it falls short of meeting the other expectations, as shown in Table 2.^12^ This implies that these tests do not solely fulfill the WHO target product profile for VL diagnostics. Additionally, in VL-endemic districts where there is a shortage of resources and skilled manpower, efficient resource use and the need to limit invasive procedures cannot be overemphasized. This underscores the importance of reducing the number of tissue aspirations and PCR tests as much as possible by using simpler and safer alternative diagnostics.

The sequential algorithm starting with the KAtex test exhibited comparable performance and helped save resources by limiting the number of patients who needed PCR testing or tissue aspiration. KAtex is cheap and simple to perform, meets the WHO target profile PPV (75%), and can be easily introduced in district areas to help achieve decentralization of care.^22^ Eligible patients can be tested at the district level and receive treatment if they test positive. Only those suspected VL patients who test negative with KAtex need PCR testing or referral to a higher level of care for subsequent evaluation.

The sequential algorithm approach fell short of the WHO target product specificity (81% versus 96.5%).^12^ The parallel testing algorithm also exhibited only little improvement in specificity (85%). Additionally, the parallel testing approach is resource-intensive, requiring all suspected patients to undergo PCR testing and leading to a higher number of tissue aspirations. Alternatively, using the semi-quantitative results of KAtex and PCR testing in the sequential algorithm yielded an improvement in specificity (91.5%), as shown in Table 3 and Supplemental Figures 1 and 2. Although this part of the analysis was affected by a small subgroup sample size, it revealed that specificity may be improved using semi-quantitative KAtex and PCR results. Adequately powered studies are needed to support this evidence. Although suboptimal specificity can reduce PPV, high pretest probability is needed to achieve it. Clinicians need to carefully select patients who need testing. On the other hand, experts may need to reconsider the feasibility of the high (96.5%) specificity requirement of the WHO target product.

Improved diagnostics and decentralization of care are important components in VL control and elimination programs.^5^^,^^23^ Implementing KAtex testing requires care and training of the primary healthcare providers who will be using it. Testing should be performed only on clinically suspected VL patients. Improper use of diagnostics can result in poor performance and unnecessary wastage of resources, as seen with rK39 RDT utilization.^24^

The clinical manifestation of VL in HIV patients can be atypical and pose additional challenges in selecting who to test. Some patients with atypical manifestations, which are common among HIV patients, might have been missed from being tested and included in the present study.

The PPV of PCR among patients who test negative via KAtex is low (28.6%). The added value of using PCR in a sequential approach is its high exclusion power (when results are negative) and its ability to minimize the number of patients who need to undergo tissue aspiration. Molecular diagnostics may not be readily available in VL-endemic regions; however, there is increasing experience with molecular diagnostics in resource-limited countries because of COVID-19, including implementation of drug-resistant tuberculosis and HIV viral load detection. Previous studies in Sudan and Ethiopia have revealed good performance of loop-mediated isothermal amplification (LAMP) for VL diagnosis.^25^^,^^26^ The practical feasibility of LAMP in low-resource settings needs to be investigated. Developing and introducing simple, user-friendly, point-of-care molecular tests for *Leishmania* diagnosis would help to decentralize molecular diagnosis. In areas where molecular diagnostics are unavailable, the proposed sequential diagnostic algorithm could include tissue aspiration after an initial KAtex result (if negative). Alternatively, samples can be shipped from remote care centers to PCR centers in a cold chain or preserved 1:1 in 2X DNA/RNA shield (Zymo Research, Irvine, CA, USA), which keeps DNA stable at 4°C to 30°C for a month, or at elevated temperatures of 35°C to 40°C for up to 7 days.

An additional potential advantage of a molecular test is its use as a test of cure. HIV-infected patients encounter frequent relapses of VL disease.^27^ There is a need for a noninvasive test of cure after a treatment course to help decide whether to administer secondary prophylaxis or extend treatment, which is currently based on visible parasitemia on tissue aspirates.^28^ Although KAtex was previously found to be inaccurate as a cure test, blood PCR testing remains to be evaluated for this indication.^13^^,^^14^ Molecular tests could also benefit immunocompetent patients with suspected VL because current algorithms still require invasive testing in those with poor treatment response.

One limitation of blood PCR testing is its specificity (64%), which is below the WHO target profile. This is most likely due to the limitation of the reference diagnostic, tissue aspiration, which is specific but less sensitive.^8^^,^^21^ The present study also revealed improved specificity of PCR testing compared with splenic aspiration, rather than with bone marrow aspiration. On the other hand, VL/HIV patients can be persistently positive on blood PCR testing for *leishmania*, even during asymptomatic periods.^6^ The sequential approach with KAtex has improved the specificity (81%), although not yet to the required target of 96.5%.^12^ Adjusting the PCR Ct cutoff value on the basis of pre-test probability (additional clinical evidence) may help to achieve the target profile and needs to be explored. The additional analysis with semi-quantitative KAtex and PCR Ct value subcategories was limited by the small sample size of the present study. However, higher PPV was noted with KAtex 3+ and PCR Ct <25.

## CONCLUSION

In summary, the East Africa region, which currently has the highest VL caseload in the world, has significant challenges in VL diagnostics. This is due to the suboptimal performance of rK39 serology in the region and the high prevalence of HIV coinfection and frequent VL relapses that render the use of antibody detection methods unhelpful.^10^ Thus, the confirmatory diagnosis for VL in HIV patients remains microscopy of tissue aspirations, which is limited to higher-care-level centers where skilled manpower and resources for the management of potential complications are available. Tissue aspiration cannot easily be decentralized for the VL elimination strategy in East Africa. Additionally, HIV-coinfected VL patients must undergo several such invasive procedures because of the frequently relapsing nature of the disease in this population, as well as the need for a test of cure.^27^ This study revealed that KAtex and blood PCR testing in the sequential algorithm can provide a useful alternative with better performance and reduced need for invasive procedures. Easy-to-use and field-applicable molecular diagnostic techniques need to be made available for use in these settings to achieve early case detection, particularly in HIV-infected population groups that might play a significant role in disease transmission.

## Supplemental Materials

10.4269/ajtmh.26-0104 Supplemental Materials
