# Supplementary material for: A Diagnostic Algorithm Based on Urine Antigen and Blood Polymerase Chain Reaction Testing for Visceral Leishmaniasis in HIV-Coinfected Patients in Northwest Ethiopia
Source: Am J Trop Med Hyg. 2026 Sep 15;115(5):989–96. doi: 10.4269/ajtmh.26-0104 (PMC13590951; doi:10.4269/ajtmh.26-0104)
Supplement: Supplementary file 1 [file tpmd260104.SD1.pdf]

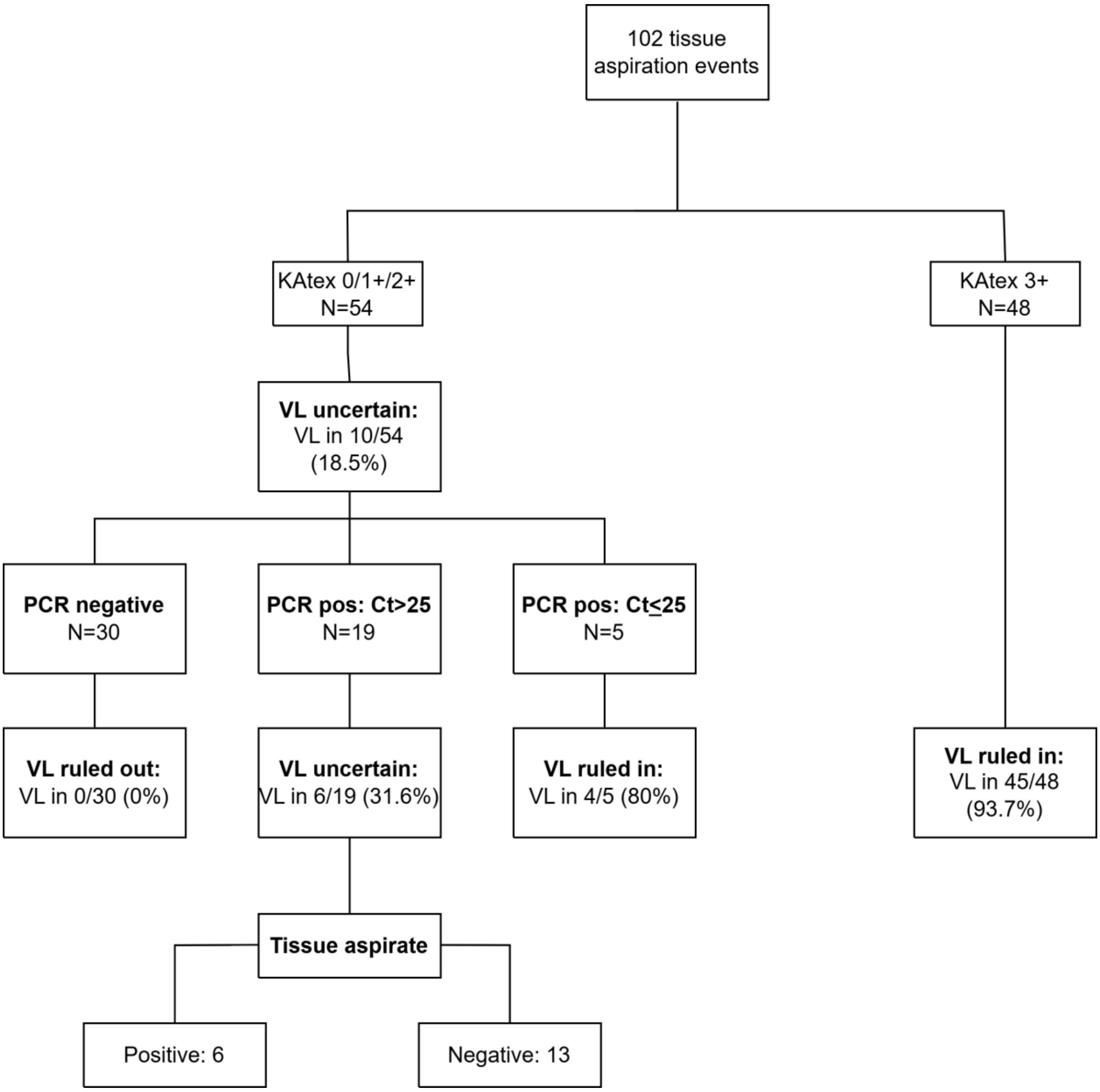

Supplemental Figure 1. Sequential diagnostic algorithm for VL in HIV coinfected patients using KAtex semi-quantitative results and PCR cycle threshold (Ct) values. VL: visceral leishmaniasis.

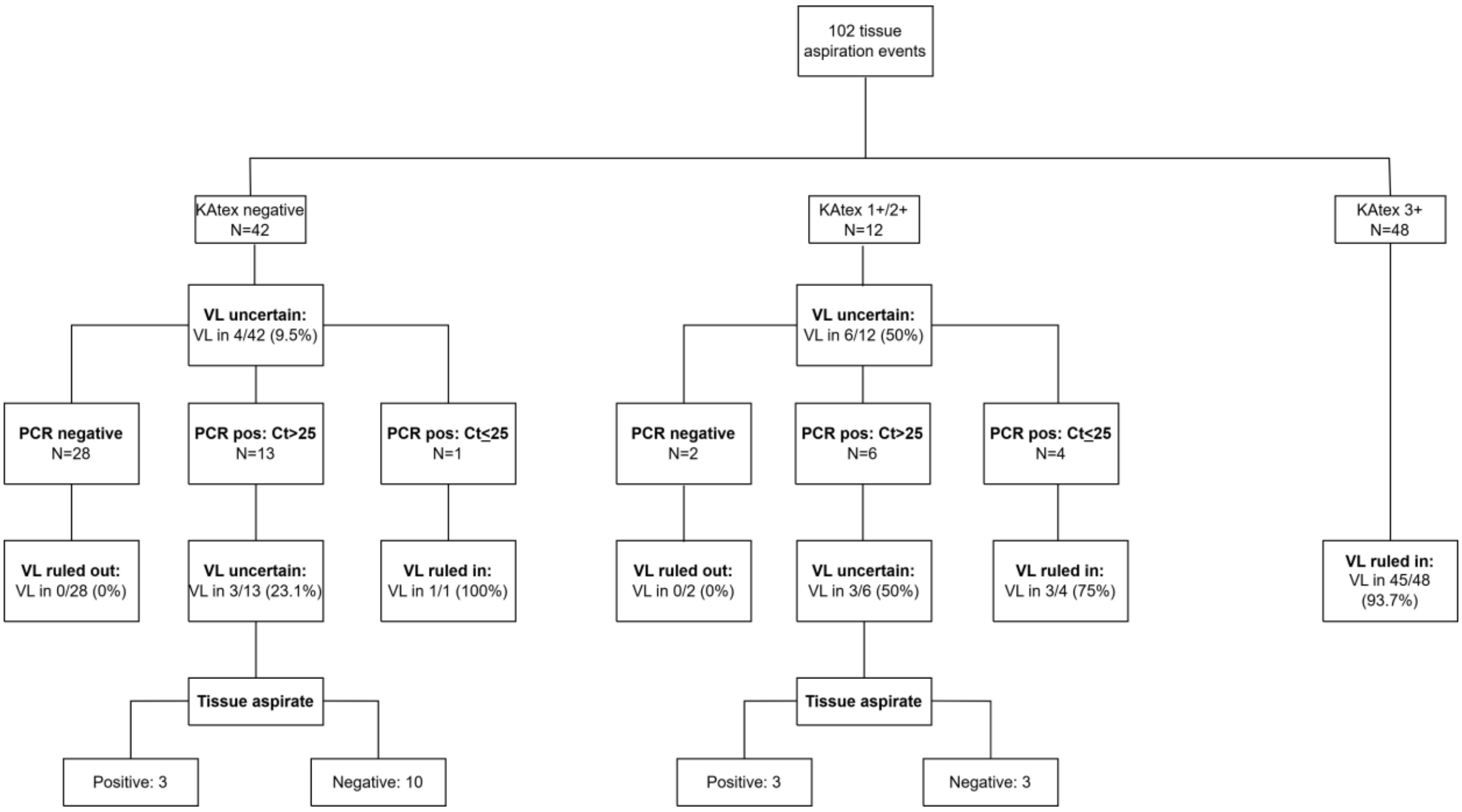

Supplemental Figure 2. Sequential diagnostic algorithm for VL in HIV coinfected patients using categorical KAtex semi-quantitative results and PCR cycle threshold (Ct) values. VL: visceral leishmaniasis.
